# Novel Therapeutic Application of Self-Assembly Peptides Targeting the Mitochondria in In Vitro and In Vivo Experimental Models of Gastric Cancer

**DOI:** 10.3390/ijms21176126

**Published:** 2020-08-25

**Authors:** Dong Jin Kim, M. T. Jeena, Ok-Hee Kim, Ha-Eun Hong, Haeyeon Seo, Ja-Hyoung Ryu, Say-June Kim

**Affiliations:** 1 Department of Surgery, Eunpyeong St. Mary’s Hospital, College of Medicine, The Catholic University of Korea, Seoul 03312, Korea; djdjcap@catholic.ac.kr; 2 Department of Chemistry, Ulsan National Institute of Science and Technology (UNIST), Ulsan 44919, Korea; jeena1991@unist.ac.kr (M.T.J.); jhryu@unist.ac.kr (J.-H.R.); 3 Department of Surgery, Seoul St. Mary’s Hospital, College of Medicine, The Catholic University of Korea, Seoul 06591, Korea; ok6201@hanmail.net (O.-H.K.); hhe49@naver.com (H.-E.H.); searcx12@naver.com (H.S.); 4 Catholic Central Laboratory of Surgery, Institute of Biomedical Industry, College of Medicine, The Catholic University of Korea, Seoul 06591, Korea

**Keywords:** Mito-FF (mitochondria-accumulating phenylalanine dipeptide with triphenyl phosphonium), 5-fluorouracil, self-assembly, reactive oxygen species, antioxidant enzymes

## Abstract

Here, we provide the possibility of a novel chemotherapeutic agent against gastric cancer cells, comprising the combination of 5-fluorouracil (5-FU) and a mitochondria-targeting self-assembly peptide, which is a phenylalanine dipeptide with triphenyl phosphonium (Mito-FF). The anticancer effects and mechanisms of 5-FU and Mito-FF, individually or in combination, were compared through both in vitro and in vivo models of gastric cancer. Our experiments consistently demonstrated that the 5-FU and Mito-FF combination therapy was superior to monotherapy with either, as manifested by both higher reduction of proliferation as well as an induction of apoptotic cell death. Interestingly, we found that combining 5-FU with Mito-FF leads to a significant increase of reactive oxygen species (ROS) and reduction of antioxidant enzymes in gastric cancer cells. Moreover, the inhibition of ROS abrogated the pro-apoptotic effects of combination therapy, suggesting that enhanced oxidative stress could be the principal mechanism of the action of combination therapy. We conclude that the combination of 5-FU and Mito-FF exerts potent antineoplastic activity against gastric cancer cells, primarily by promoting ROS generation and suppressing the activities of antioxidant enzymes.

## 1. Introduction

The lack of anticancer chemotherapeutic agents is a fundamental issue faced while treating advanced gastric cancer. Therapeutic failure to effectively eradicate gastric cancer cells is mostly caused by the development of drug resistance. The mechanisms by which cancer cells develop drug resistance include drug inactivation, alteration in drug targets, adaptive responses, and dysfunctional apoptosis, all of which are due to the adaptive genetic mechanisms of cancer cells [1]. Therefore, overcoming drug resistance depends, to a considerable extent, on finding a way to evade these genetic surveillance mechanisms of tumor cells.

Mitochondrion is one of the distinguished structures unique to eukaryotic cells. It principally serves as an energy source, enabling numerous functions of the cell. Though mitochondria are a part of the cell, they were previously independent organisms unrelated to the genetic mechanisms of the host cells. Therefore, it is worthwhile to consider targeting mitochondria with the purpose of evading the surveillance of a tumor’s genetic mechanisms that culminate in drug resistance. 

We previously validated the pronounced anticancer properties of a newly developed mitochondria-accumulating phenylalanine dipeptide with triphenyl phosphonium (Mito-FF) [2]. Mito-FF is a synthetic compound that is composed of diphenylalanine (FF) and triphenylphosphonium (TPP), with pyrene as a fluorescent probe. Diphenylalanine has the potential to self-assemble into well-ordered tubular structures. It can have an elongated length of up to 10 µm, by forces arising from hydrogen bonds and π–π stacking interactions of aromatic residues [3]. TPP is a delocalized lipophilic cation; therefore, it is particularly effective at crossing the hydrophobic inner mitochondrial membrane, resulting in >1000-fold accumulation within the mitochondrial matrix [4]. Therefore, Mito-FF supposedly selectively accumulates into mitochondria, and therein destructs mitochondria by transforming into stiff Mito-FF fibrils. We previously showed that Mito-FF predominantly accumulates inside the mitochondria and arrives at the critical aggregation concentration to establish a fibrous nanostructure, culminating in mitochondrial disruption and subsequent apoptosis [2]. In this study, we attempted to determine the anticancer activities of Mito-FF against gastric cancer cells and sought to find a way of potentiating these activities.

## 2. Results

### 2.1. Cell Viability Assay in Normal Gastric Cells and Gastric Cancer Cells

**Figure 1:**
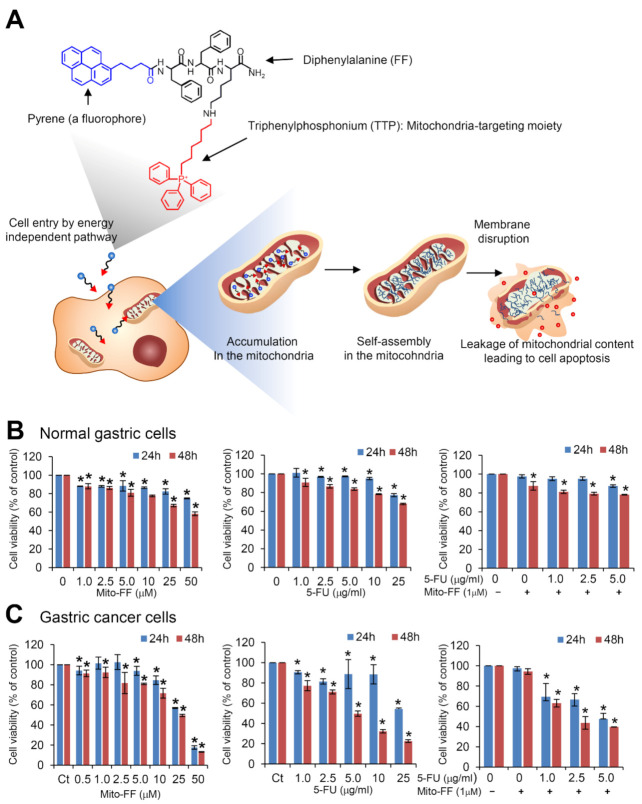
Action mechanism of triphenyl phosphonium (Mito-FF) and cell viability assay of 5-fluorouracil (5-FU) and Mito-FF, individually and in combination. (**A**) Intra-mitochondrial assembly of mitochondria-accumulating phenylalanine dipeptide with triphenyl phosphonium (Mito-FF). Mito-FF consists of diphenylalanine as a β-sheet-forming building block, triphenylphosphonium (TPP) as a mitochondrial targeting moiety, and pyrene as a fluorescent probe. Cancer cells have higher mitochondrial membrane potential, which leading to a higher accumulation of Mito-FF in the mitochondria, followed by self-assembly into fibrils within mitochondria. The self-assembled fibrils promote the disruption of the mitochondrial membrane, resulting in a leakage of mitochondrial contents into the cytosol, eventually inducing cellular apoptosis. (**B**) Cell viability test of Mito-FF (Left), 5-FU (Middle), and the combination of 5-FU and Mito-FF in normal gastric cells (Right). 5-FU and Mito-FF induced a dose- and time-dependent reduction of viability in normal gastric cells. In addition, combining these two materials induced an additive reduction of viability in normal gastric cells. (**C**) Cell viability test of Mito-FF (Left), 5-FU (Middle), and the combination of 5-FU and Mito-FF in AGS cells. Combining these two materials resulted in the prominent reduction of viability in AGS cells, which is suggestive of a synergistic effect. Values are presented as mean ± standard deviation of three independent experiments. * *P* < 0.05.

Mito-FF consists of diphenylalanine as a β-sheet-forming building block, triphenylphosphonium (TPP) as a mitochondrial targeting moiety, and pyrene as a fluorescent probe (Figure 1A). It was designed to induce cancer cell apoptosis through mitochondrial disruption by accumulation around the mitochondria of cancer cells, penetration into the mitochondrial matrix across the mitochondrial membrane, and self-assembly into fibrils. 

We first compared cell viability according to mono- and combination therapy using 5-fluorouracil (5-FU) and Mito-FF in both human primary stomach epithelial cells and AGS cells. We herein used low concentration (1 μM) of Mito-FF to minimize toxicity to the cells. In normal gastric cells, combining 5-FU and Mito-FF induced additive reduction of viability in normal gastric cells (Figure 1B). However, in adeonocarcinoma gastric (AGS) cells, we found that combining these two materials resulted in a prominent reduction of viability (Figure 1C).

### 2.2. Cell Apoptosis Assay in Gastric Cancer Cells

**Figure 2:**
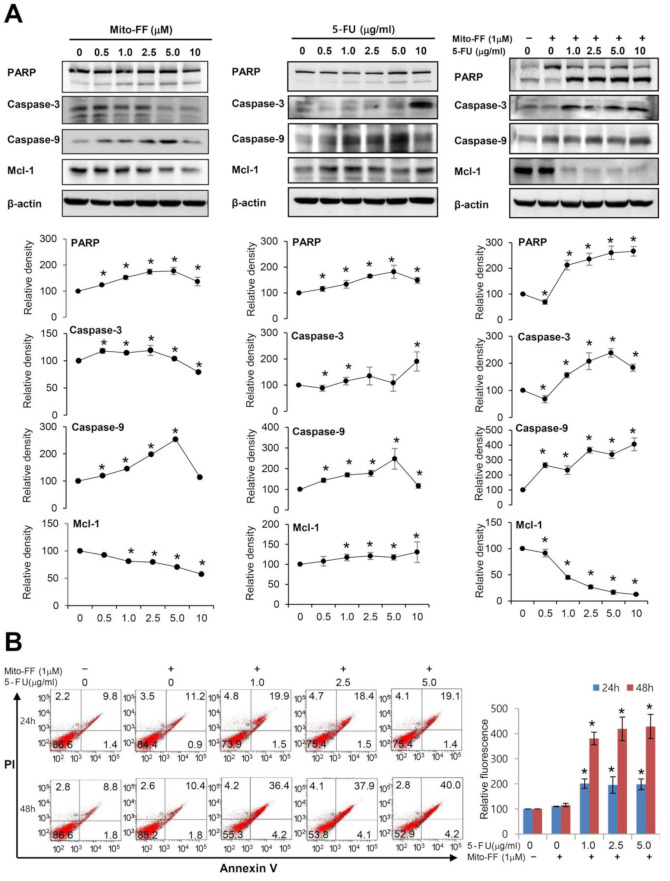
Cell apoptosis assay in gastric cancer cells. (**A**) Western blot analysis to determine the changes of pro-apoptotic (poly-ADP (adenosine diphosphate)-ribose polymerase (PARP), caspase-3, and caspase-9) and an anti-apoptotic (myeloid cell leukemia-1 (Mcl-1)) marker according to mono- and combination therapy using 5-FU and Mito-FF in adeonocarcinoma gastric (AGS) cells. In all treatments, as the concentrations were increased, pro-apoptotic markers increased, whereas anti-apoptotic markers decreased. These trends were most prominent in combination therapy. (**B**) Determination of apoptosis by flow cytometric analysis of Annexin V/propidium iodide staining [Left]. The proportion of apoptosis is determined by the total fraction of Annexin V-positive cells (the combination of early and late apoptotic cells). [Right] Relative percentages of apoptotic cells following each treatment. The number of Annexin V-positive cells was significantly increased following combining mito-FF with increasing concentration of 5-FU, especially 48 h after treatment. Values are presented as mean ± standard deviation of three independent experiments. * *P* < 0.05.

We performed a Western blot analysis to determine the changes of pro-apoptotic (poly-ADP (adenosine diphosphate)-ribose polymerase (PARP), caspase-3, and caspase-9) and anti-apoptotic (Mcl-1) markers according to mono- and combination therapy using 5-FU and Mito-FF in AGS cells (Figure 2A). As the concentrations of 5-FU and Mito-FF were raised, the pro-apoptotic markers increased up to a certain concentration, and the anti-apoptotic marker Mcl-1 decreased. Mito-FF monotherapy caused severe cell damage at concentrations above 5 µM. In addition, 5-FU monotherapy caused severe cell damage at concentrations above 5 µg/mL. However, Mito-FF and 5-FU combination therapy showed rapid cell damage according to the increasing concentration of 5-FU even at a low concentration of Mito-FF (1 µM). Therefore, it appears that Mito-FF and 5-FU combination therapy synergistically promotes apoptotic cell death of AGS cells.

Subsequently, the pro-apoptotic effects of each treatment modality were further validated by quantitatively measuring apoptosis, which is manifested by the proportion of Annexin V-positive cells, using Annexin V/propidium iodide (PI) staining and flow cytometry (Figure 2B). It was found that the proportion of apoptotic cells was significantly increased following combining mito-FF with increasing concentration of 5-FU, especially 48 h after treatment (*P* < 0.05).

### 2.3. Anticancer Activities of Each Treatment in Relation with Oxidative Stress

**Figure 3:**
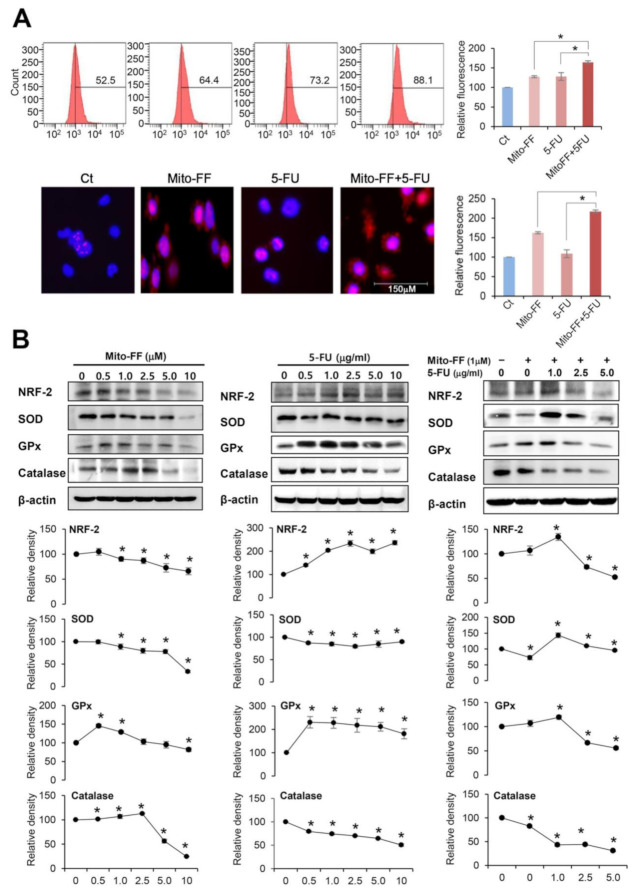
Anticancer activities of each treatment in relation with oxidative stress. (**A**) [Top] Estimation of superoxide levels in AGS cells by MitoSOX-based flow cytometric assay. [Bottom] Quantification of superoxide levels in AGS cells as determined by MitoSOX immunostaining (red signal). Collectively, combination therapy significantly enhanced the intensity of fluorescence of the AGS cells to bright red far more than monotherapy, suggesting the superiority of combination therapy over monotherapy in promoting mitochondrial superoxide synthesis. (**B**) Western blot analysis to determine the changes of NRF-2 and antioxidant enzymes (superoxide dismutase (SOD), glutathione peroxidase (GPx), and catalase) according to mono- and combination therapy using 5-FU and Mito-FF in AGS cells. As the concentrations were increased, monotherapy (either 5-FU or Mito-FF) led to the reduced expression of NRF-2 and antioxidant enzymes. Moreover, combination therapy induced the most prominent reduction of expression in these markers. Values are presented as mean ± standard deviation of three independent experiments. * *P* < 0.05.

Chemotherapeutic agents can have the potential of generating higher amount of reactive oxygen species (ROS) while significantly reducing the expression of antioxidant enzymes [5,6]. Therefore, we investigated the effects of 5-FU and mito-FF, individually or in combination, on the intracellular ROS levels of AGS gastric cancer cells. MitoSOX Red reagent (a red mitochondrial superoxide indicator) is oxidized by superoxide in the mitochondria to produce red fluorescence [7]. We determined intracellular ROS levels by MitoSOX-based flow cytometric assay as well as by MitoSOX staining, of which the fluorescence intensity of MitoSOX is known to be proportional to the cytosolic ROS levels (Figure 3A). Combination therapy enhanced the fluorescence intensity of the AGS cells to the color with bright red more than individual monotherapies did (*P* < 0.05), suggesting the superiority of combination therapy over monotherapy in promoting mitochondrial ROS synthesis. 

Next, we performed a Western blot analysis to determine the changes of nuclear factor erythroid 2-related factor 2 (NRF-2) (a cellular sensor of oxidative stress) and antioxidant enzymes (superoxide dismutase (SOD), glutathione peroxidase (GPx), and catalase) according to mono- and combination therapy using 5-FU and Mito-FF in AGS cells (Figure 3B). As the concentration of Mito-FF increased, the expression of both NRF-2 and antioxidant enzymes tended to decrease. In addition, as the concentration of 5-FU increased, the expression of NRF-2 increased, and the expression of antioxidant enzymes tended to decrease. Moreover, Mito-FF and 5-FU combination therapy resulted in the significant reduction of the expression of both NRF-2 and antioxidant enzymes, which is suggestive of their synergistic effect.

### 2.4. Effects of Combination Therapy on AGS Cell Growth Xenografted in Nude Mice

**Figure 4:**
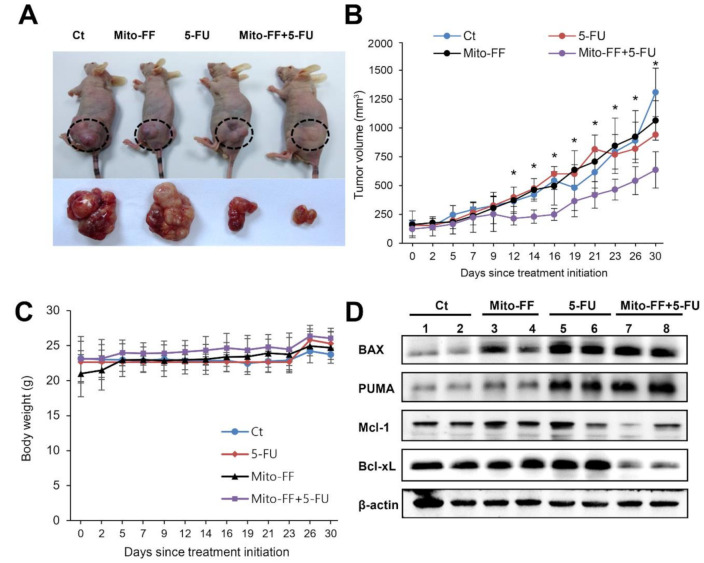
Effects of combination therapy on AGS cell growth xenografted in nude mice. The nude mice were intraperitoneally injected with 5-FU (1.25 mg/kg), Mito-FF (50 µg/kg), or both 3 times a week for 4 weeks. Subsequently, the tumors in the mice were collected after euthanizing them. (**A**) Photographs showing gross morphology of mice with xenografted AGS cells in each group. The reduction of tumors was most prominent in the mice treated with combination therapy, which was followed by 5-FU and Mito-FF treatments. (**B**) Tumor size after each treatment. Tumor size refers to the longest axis of the extracted tumor. Combination therapy induced the most significant reduction in the tumor size. (**C**) Body weight changes during experiment. The average body weight of mice in each group did not vary significantly over the course of the experiment. (**D**) Western blot analyses of tumor specimens in each group. Combination therapy most significantly increased the expression of a pro-apoptotic marker (Bax and PUMA) and decreased the expression of anti-apoptotic markers (Mcl-1 and Bcl-xL). Values are presented as mean ± standard deviation of three independent experiments. * *P* < 0.05.

We validated the effects of mono- or combination therapy on the growth of AGS cells xenografted in nude mice. Male BALB/c nude mice (5 weeks old) were used for the comparative modeling of subcutaneous tumor growth. AGS cells (10^7^) were subcutaneously injected into each mouse. Three weeks after tumor cell injection, all mice had measurable tumors. Then, mice were randomly grouped (*n* = 10 per group) and treated intraperitoneally with normal saline (control), 5-FU (1.25mg/kg, 3 times a week), Mito-FF (50μg/kg, 3 times a week), and a combination of both agents (3 times a week) for 30 days, respectively. Immediately after the completion of 30-day administration, the mice were euthanized, which was subsequently followed by attainment of their tumors. Images of the tumors before and after necropsy showed that the shrinkage was most prominent in mice treated with combination therapy, followed by 5-FU and, subsequently, Mito-FF (Figure 4A). Combination therapy induced the most significant reduction in tumor size (*P* < 0.05) (Figure 4B). However, there was no difference in the average body weight of mice between each group (Figure 4C). Western blot analysis of the specimens showed that combination therapy most significantly increased the expression of pro-apoptotic markers (bcl-2-like protein 4 (Bax) and nuclear factor erythroid 2-related factor 2 (PUMA)) and decreased the expression of anti-apoptotic markers (Mcl-1 and Bcl-xL) (Figure 4D). 

### 2.5. Determination of Antioxidant Activities of Individual Treatment in the Xenografted AGS Cells

**Figure 5:**
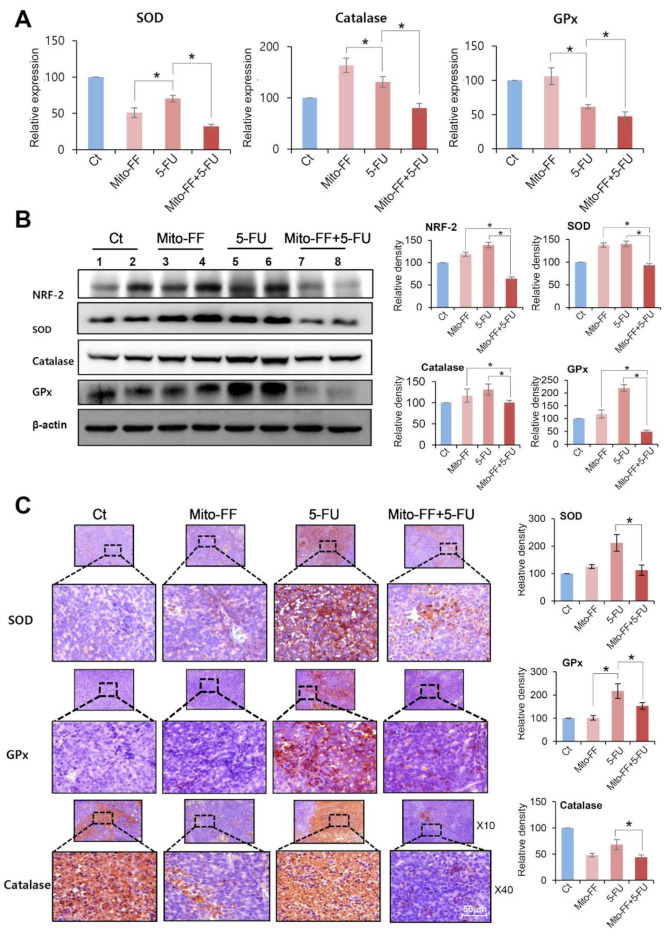
Determination of antioxidant activities of individual treatments in the xenografted AGS cells. (**A**) Real-time PCR using specimens obtained from xenografted AGS cells following individual treatments. Combination therapy most significantly reduced the RNA expression of antioxidant enzymes, including catalase, SOD, and GPx compared to monotherapy. (**B**) Western blot analyses using specimens obtained from xenografted AGS cells following individual treatments. Combination therapy most significantly reduced the expression of NRF-2 and various antioxidant enzymes compared to monotherapy. (**C**) Immunohistochemical stains demonstrating that combination therapy most significantly reduced the expression of antioxidant enzymes, including SOD, catalase, and GPx compared to the monotherapy. Values are presented as mean ± standard deviation of three independent experiments. * *P* < 0.05.

We intended to determine antioxidant effects of each treatment in the in vivo tumor xenograft model. Then, we performed real-time PCR and Western blot analysis using xenografted AGS cells. Real-time PCR showed that combination therapy most significantly reduced the expression of antioxidant enzymes, such as SOD, catalase, and GPx compared to monotherapy with either 5-FU or Mito-FF (*P* < 0.05) (Figure 5A). Western blot analysis validated the consistent results as did real-time PCR, as well as significantly reduced the expression of NRF-2 in the specimens treated with combination therapy (*P* < 0.05) (Figure 5B).

Subsequently, we performed immunohistochemical staining using xenografted AGS cells to reaffirm the antioxidant activities of individual therapies. Immunohistochemical stains reaffirmed that combination therapy most significantly reduced the expression of antioxidant enzymes, such as SOD, catalase, and GPx compared to monotherapy with either 5-FU or Mito-FF (*P* < 0.05) (Figure 5C).

### 2.6. Inhibition Test for the Determination of the Action of Combination Therapy

**Figure 6:**
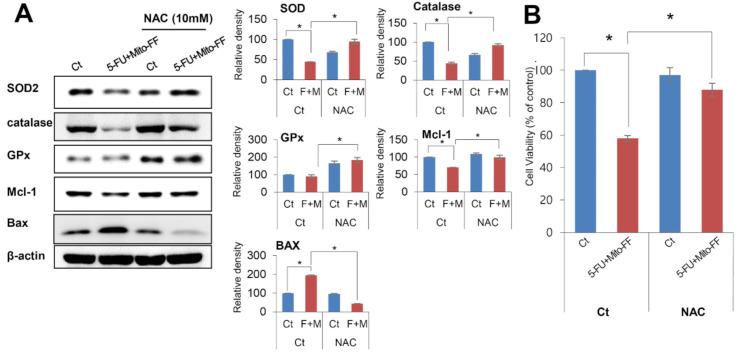
Inhibition test for the determination of the mechanism of action of combination therapy. (**A**) [Left] The effects of reactive oxygen species (ROS) inhibition on the expression of proteins related with antioxidant activity and apoptosis in AGS cells treated with combination therapy (5-FU plus Mito-FF). N-acetyl-L-cysteine (NAC) was used as a ROS inhibitor. [Right] Relative densities of these markers in each group. Although combination therapy led to the lower expression of antioxidant enzymes (SOD, catalase, and GPx) as well as pro-apoptotic tendencies, which were expressed by the higher expression of a pro-apoptotic marker (Bax) and the lower expression of an anti-apoptotic marker (Mcl-1), these effects were significantly abrogated by NAC. (**B**) Effects of ROS inhibition on the cell proliferation of AGS cells treated with combination therapy. NAC significantly abrogated the anti-proliferative effects of combination therapy (*P* < 0.05).

To determine anticancer effects of combination therapy in relation with oxidative stress, we performed an ROS inhibition test using N-acetyl-L-cysteine (NAC), which is an ROS inhibitor [8]. Specifically, we collected the tumor specimens treated with combination therapy and performed Western blot analyses demonstrating the expression of antioxidant enzymes with or without NAC (Figure 6A). As previously shown, combination therapy led to the lower expression of antioxidant enzymes as well as pro-apoptotic tendencies, which were expressed by the higher expression of pro-apoptotic markers and the lower expression of anti-apoptotic markers. We found that the ROS inhibition by NAC abrogated these effects of combination therapy. NAC increased the expression of antioxidant enzymes (SOD, catalase, and GPx) and decreased pro-apoptotic tendencies, which was manifested by the decreased expression of a pro-apoptotic marker (Bax) and increased expression of an anti-apoptotic marker (Mcl-1). Proliferation assay showed that NAC abrogated the anti-proliferative effects of combination therapy in AGS cells (*P* < 0.05) (Figure 6B). Taken altogether, our results suggest that the mechanism of action of the combination therapy could be attributed to the enhanced oxidative stress. 

## 3. Discussion

In this study, we aimed to determine whether the combination of 5-FU and Mito-FF has superior anticancer effects against gastric cancer over individual monotherapies in terms of inducing apoptotic cell death and promoting oxidative stress. Our in vitro and in vivo experiments consistently demonstrated that the 5-FU and Mito-FF combination therapy was superior to the monotherapies. This was manifested by both a higher reduction of proliferation and the induction of apoptosis. Mito-FF is a synthetic material, which selectively targets mitochondria and disrupts them during its process of self-assembly. As expected, Mito-FF led to the increase of intracellular ROS production and a decreased expression of antioxidant enzymes. Interestingly, we found that combining 5-FU with Mito-FF potentiated Mito-FF-induced oxidative stress. The inhibition of ROS by NAC abrogated the pro-apoptotic effects of combination therapy, demonstrating that upregulated oxidative stress could be the principal mechanism of the action of combination therapy. Therefore, our results suggest that this combination provides a novel therapeutic approach for the eradication of gastric cancer cells, and further studies are required to determine the clinical potential of this combination therapy.

The mitochondrion is one of the essential organelles that determine the continued survival or death of cells. The first and essential role of the mitochondrion is to generate adenosine triphosphate (ATP), the energy source of the cell, which accompanies a continuous flow of electrons by way of the oxidative phosphorylation process. Furthermore, mitochondria are the major source of ROS that are generated as by-products of the electron transport chain [4]. Oxidative stress is caused by an excessive amount of ROS, which is a consequence of either increased ROS generation, reduced activity of antioxidant enzymes, or both. Therefore, mitochondrial dysfunction is detrimental to the cell, because it can be directly related with a lack of energy as well as excessive oxidative stress. Thus, it is reasonable to target mitochondria with the purpose of eradicating the cells containing them.

Molecular self-assembly is a spontaneous process of generating ordered structures under specific thermodynamic and kinetic conditions, arising from specific and local molecular interactions [9,10]. It is orchestrated either physiologically or pathologically, and it is considered one of the most essential and fundamental biological systems. Examples of biological self-assembly includes the formation of the DNA double helix through hydrogen bonding, the assembly of biological membranes comprising of phospholipids, the association of protein microtubules and microfilaments to provide cellular support, and the formation of amyloid fibrils in patients with Alzheimer’s disease [3,11]. Inspired by nature, investigators have generated a number of self-assembly biomimetic materials for various purposes [12,13,14,15,16,17,18]. We generated Mito-FF with the aim of attacking tumor cells while circumventing the possibility of developing drug resistance. 

Mito-FF has several characteristics. Firstly, the diphenylalanine peptide was used as the peptide building block for self-assembly in Mito-FF. Diphenylalanine peptide is an essential building block in the amyloid assembly process in Alzheimer’s and other neurodegenerative diseases. Through self-assembly powered by hydrogen bonding and π–π stacking of aromatic residues, it can be organized into well-ordered tubular structures with a long persistence length [3,19,20,21,22]. It subsequently leads to cytotoxicity due to the formation of noxious fibril aggregates [23]. Secondly, the incorporation of TPP enables Mito-FF to specifically accumulate around mitochondria. TPP is a lipophilic cation that can easily permeate across mitochondrial membranes, as it is a positively charged compound and the mitochondrial inner membrane has a negative membrane potential. Subsequently, TPP accumulates in the mitochondrial matrix [24]. Finally, pyrene, although not an essential element in Mito-FF, was tagged as an experimental identification marker. 

The preferential binding of Mito-FF into cancer cells can be explained by the higher negative membrane potential of cancer cells than that of normal cells [25,26]. Cancer cells have a much lower cell membrane potential (approximately –180 to –220 mV) than that (120–160 mV) of normal cells due to excessive glycolysis. Thus, Mito-FFs preferentially approach cancer cells because they contain TPP, which is a delocalized lipophilic cation [4]. In addition, cancer cells usually have dysfunctional mitochondria, which are unable to meet their energy needs. Accordingly, the mitochondrial membrane potentials of cancer cells become more negative, because mitochondrial dysfunction causes the dissipation of a fraction of the mitochondrial proton gradient. Thus, positively charged Mito-FFs in the cancer cells preferentially approach the mitochondria with more negative membrane potentials, leading to a higher accumulation of Mito-FF in the mitochondria, followed by self-assembly into fibrils within mitochondria. The self-assembled fibrils promote the disruption of the mitochondrial membrane, resulting in the leakage of mitochondrial contents into the cytosol, eventually inducing cellular apoptosis.

In this study, we found that that the 5-FU and Mito-FF combination therapy was superior to monotherapy with either individually, as manifested by both a higher reduction of proliferation and a higher induction of apoptotic cell death as well. Mito-FF preferentially accumulates inside the mitochondria and reaches the critical aggregation concentration to form a fibrous nanostructure, culminating in mitochondrial disruption and subsequent apoptosis. Thus, Mito-FF was expected to exert its anticancer effects primarily by promoting oxidative stress, which is manifested by both the upregulation of ROS and downregulation of antioxidant enzymes. Although Mito-FF had the potential of promoting oxidative stress, it appeared not to too be potent as anticipated. However, we also found that enhancement of oxidative stress by Mito-FF is potentiated by the addition of 5-FU; the addition of 5-FU to Mito-FF led to the remarkable outcomes in terms of a higher upregulation of ROS and downregulation of antioxidant enzymes as well.

5-FU is predominantly used to treat solid cancers, including gastrointestinal, pancreatic, head, neck, and breast cancers. 5-FU exerts its anticancer effects by interrupting the function of thymidylate synthase. This interruption blocks the synthesis of thymidine that belongs to pyrimidine nucleoside required for DNA replication [27]. Recently, several investigators reported that 5-FU exerts its anticancer effects, in part, by increasing intracellular oxidative stress [27,28,29]. The activities of SOD and glutathione peroxidase were lowered in 5-FU-treated guinea pigs, demonstrating a reduced antioxidant capacity [28]. The cause of 5-FU-induced cardiotoxicity is thought to be the increased intracellular levels of ROS by 5-FU [29]. Lamberti et al. also showed that 5-FU induces apoptosis in rat cardiocytes through enhancing intracellular oxidative stress [29]. In our study, although 5-FU did not exert outstanding ROS-generating potential in gastric cancer cells, it synergistically potentiated the oxidative stress by Mito-FF.

In conclusion, we found that the combination of 5-FU and Mito-FF is superior to monotherapy with either, as manifested by both a higher reduction of proliferation and a higher induction of apoptotic cell death as well. Mito-FF is a synthetic material that selectively targets and disrupts the mitochondria of cancer cells and exerts its anticancer activities by promoting oxidative stress. In our study, we obtained validated evidence that 5-FU synergistically potentiates the oxidative stress exerted by Mito-FF. Therefore, we think it is reasonable and recommendable to combine Mito-FF with 5-FU for the purpose of eradicating gastric cancer cells. Further studies are required to determine the clinical potential and possible toxicities of this combination.

## 4. Materials and Methods 

### 4.1. Chemicals and Antibodies

Mito-FF was generated in the laboratory as previously described [2]. N-acetyl-L-cysteine (NAC) was purchased from Sigma-Aldrich (St. Louis, MO). For Western blot analyses, primary antibodies were purchased from Cell Signaling Technology (Danvers, MA, USA). They included the antibodies against PUMA (nuclear factor erythroid 2-related factor 2), Bcl-XL (B-cell lymphoma-extra large), myeloid cell leukemia-1 (Mcl-1), BAX (bcl-2-like protein 4), cleaved caspase-3 (c-caspase-3), cleaved caspase-9 (c-caspase-9), cleaved poly-ADP (adenosine diphosphate)-ribose polymerase (c-PARP), superoxide dismutase (SOD), glutathione peroxidase (GPx), catalase, nuclear factor erythroid 2-related factor 2 (NRF-2), and β-actin.

### 4.2. Cell Culture and Cell Viability Assay

Human primary stomach epithelial cells (normal gastric cells) were obtained from Cellbiologics (Chicago, IL). Adeonocarcinoma gastric (AGS) cell lines were obtained from the Korean cell line bank (KCLB, Seoul, Republic of Korea). Human primary stomach epithelial cells were cultured in an Epithelial cell medium/w kit (Cellbiologics, Chicago, IL, USA), and AGS cells were cultured in RPMI (Thermo, Carlsbad, CA) with 10% fetal bovine serum (Hyclone, Logan, LT, USA), penicillin (100 U/ml), and streptomycin (100  μg/ml) (Gibco, Carlsbad, CA, USA). Cell viability assay was performed using the EZ-Cytox Cell viability Assay kit (Itsbio, Seoul, South Korea) according to the manufacturer’s instruction. 

### 4.3. Western Blot Analysis

We performed Western blot analyses using both cell lines and animal tissues as previously described [30]. 

### 4.4. MitoSOX Staining

AGS cells were cultured on Lab-Tek chamber slides (Thermo Fisher Scientific, Waltharm, MA, USA). The cells were treated with 5-FU (5-flurouracil: Sigma-Aldrich) and Mito-FF for 24 h. Subsequently, AGS cells were stained with 10 μM MitoSOX (Thermo) reagent at 37 °C for 10 minutes. The MitoSOX Red probe was observed using a fluorescence imaging system (EVOS U5000; Invitrogen, CA, USA). 

### 4.5. Quantification of Reactive Oxygen Species (ROS) and Apoptosis

Cells were stained with either MitoSOX or Annexin V/PI apoptosis detection kit (BD Biosciences) according to the manufacturer’s instruction, respectively. Subsequently, quantification of ROS and apoptosis of the cells was performed by flow cytometric analysis.

### 4.6. Real-Time Quantitative PCR

The total RNA of mouse tissues was extracted using TRIzol reagent (Invitrogen, Carlsbad, CA, USA) according to the manufacturer’s instructions. cDNA was prepared using ReverTraAce®qPCR-RT master mix (TOYOBO, OSAKA, JAPAN). Quantitative RT-PCR was performed using ThunderBird® STBR® qPCR mix (TOYOBO). The primers used for q RT-PCR were as follows: SOD forward 5’-TGGGGACAATACACAAGGCTGT-3’ and reverse 5’-TTTCCACCTTTGCCCAAGTCA-3’; Catalase forward 5’-CCTCCTCGTTCAGGATGTGGTT-3’ and reverse 5’-CGAGGGTCACGAACTGTGTCAG-3’; GPx forward 5’-CCGGGACTACACCGAGATGAA-3’ and reverse 5’-CACCAGGTCGGACGTACTTGAG-3’; GAPDH, forward 5′-CGACTTCAA-CAGCAACTCCCACTCTTCC-3′ and reverse 5′-TGGGTGG-TCCAGGGTTTCTTACTCCTT-3′ [31,32,33]. 

The qRT-PCR was performed on a Step one plus real-time PCR system (Life Technologies, Carlsbad, CA) equipped with a 96-well optical reaction plate. After being normalized to the GAPDH gene, the expression levels for each target gene were calculated using the comparative threshold cycle method. Data were presented as the mean ± standard deviation (SD) from three independent experiments.

### 4.7. In Vivo Xenograft Model

Male BALB/c nude mice (5 weeks old; OrientBio, Republic of Korea) were used for the comparative modeling of subcutaneous tumor growth. All of the surgical animal care was provided in accordance with the Laboratory Animals Welfare Act, the Guide for the Care and Use of Laboratory Animals, and the Guidelines and Policies for Rodent Survival Surgery provided the IACUC (Institutional Animal Care and Use Committee) in the school of medicine, the Catholic University of Korea (Approval number: CUMC-2018-0173-03). We generated the mouse model of xenografted gastric cancer as previously described [30]. Briefly, AGS cells (10^7^ cells) were subcutaneously injected into each mouse [34,35]. The mice were weighed twice a week. Fourteen days after tumor cell injection, all the mice had measurable tumors. Mice were selected for treatment when the subcutaneous tumors reached 3.0–20 mm diameter, as measured with a digital caliper. A 20-mm tumor diameter was selected as the maximum treatment size for the balanced comparison. Subsequently, the mice were randomly subdivided into four groups (n = 10 per group) and were intraperitoneally injected with normal saline (control), 5-FU (1.25mg/kg, 3 times a week), Mito-FF (50μg/kg, 3 times a week), and a combination of both agents (3 times a week) for 30 days, respectively. Before harvesting the tumors, mice were anesthetized with 2% isoflurane in 2 L/min oxygen. Tumor size was measured twice weekly via caliper by determining the longest axis of the extracted tumor. Subsequently, tumor volume was calculated using the formula length × width^2^ × 0.5236 [36]. Immediately after the completion of treatment, all mice were euthanized using inhalation methods using CO_2_ gas with the verification of euthanasia by cervical dislocation, and specimens were obtained for further evaluation.

### 4.8. Immunohistochemical Analyses

Formalin-fixed paraffin-embedded (FFPE) tissue were sectioned with an automated rotary microtome (Leica RM2255: Germany). Sectioned tissues were deparaffinized, rehydrated in an ethanol series, and subjected to epitope retrieval using standard procedures. Antibodies to SOD, GPx, and catalase were used for immunohistochemical stains. The sample was observed using a Pannoramic disital slide scanners system (3D HISTECH; Budapest, Hungary). 

### 4.9. Statistical Analysis

All data were presented as mean ± standard deviation (SD). Statistical comparison among groups was accomplished using the Kruskal–Wallis test followed by Dunnett’s test as the post hoc analysis. Probability values of *P* < 0.05 were regarded as statistically significant. All data were analyzed with SPSS 11.0 software (SPSS Inc., Chicago, IL, USA). 

## Abbreviations

Mito-FF	Mitochondria-accumulating phenylalanine dipeptide with triphenyl phosphonium
ROS	Reactive oxygen species
5-FU	5-Flurouracil
NAC	N-acetyl-L-cysteine
AGS	Adeonocarcinoma gastric cell
NRF-2	Nuclear factor erythroid 2-related factor 2
SOD	Superoxide dismutase
GPx	Glutathione peroxidase
c-PARP	Cleaved poly-ADP (adenosine diphosphate)-ribose polymerase; c-caspase-3: cleaved caspase-3
c-caspase-9	cleaved caspase-9
Mcl-1	myeloid cell leukemia-1
PUMA	nuclear factor erythroid 2-related factor 2
BAX	bcl-2-like protein 4
Bcl-XL	B-cell lymphoma-extra large
DMSO	dimethyl sulfoxide
HO-1	heme oxygenase 1
